# Supplementary material for: Crowdsourced biodiversity monitoring fills gaps in global plant trait mapping
Source: Nat Commun. 2026 Jan 30;17:1203. doi: 10.1038/s41467-026-68996-y (PMC12858941; doi:10.1038/s41467-026-68996-y)
Supplement: Supplementary file 1 — Supplementary Information [file 41467_2026_68996_MOESM1_ESM.pdf]

Supplementary Information

Supplementary Figures

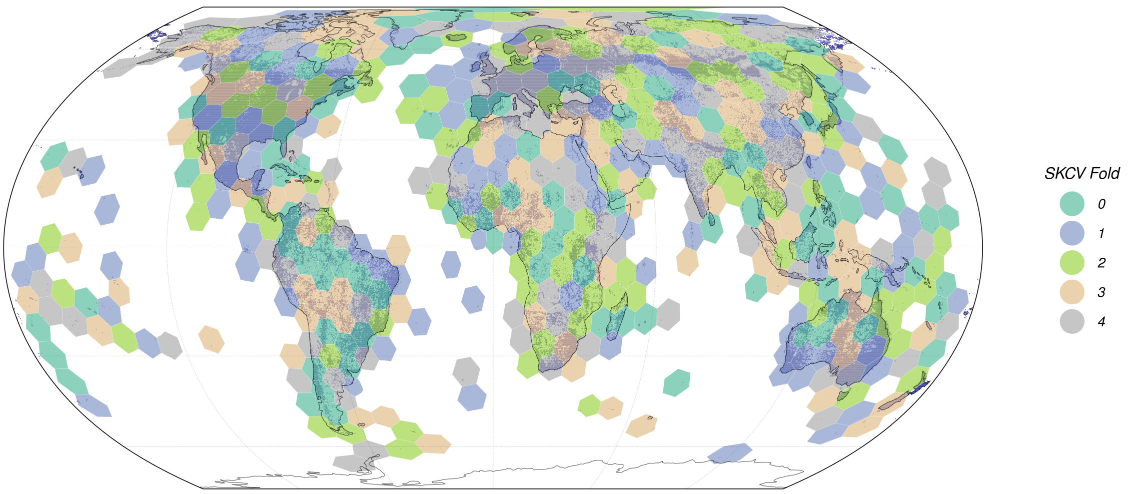

Figure S.1: **Example of spatial cross-validation folds for Leaf N (area) using the COMB trait data subset.** Dark blue points represent observations, and multicolored hexagons represent the spatial fold assignments of the points. Source data are available at `https://zenodo.org/records/18108765`.

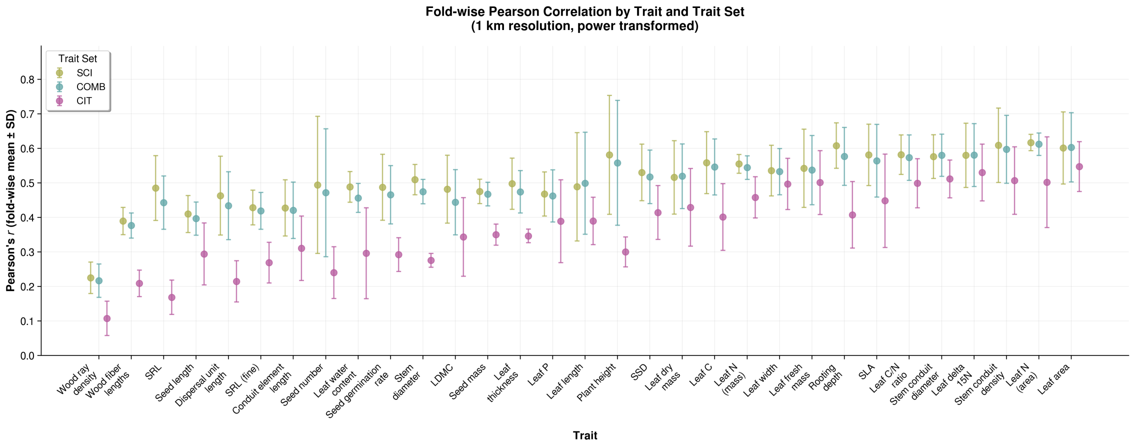

Figure S.2: **Fold-wise predictive performance of plant functional trait models across trait sets.** Fold-wise mean Pearson correlation coefficients (± standard deviation, n=5 folds) for 31 traits predicted using Earth observation data at 1 km^2 resolution. Models were trained on three trait data subsets: SCI (vegetation surveys only), COMB (combined vegetation surveys and citizen science observations), and CIT (citizen science observations only). Performance was assessed via spatial 5-fold cross-validation and therefore differs slightly from the aggregate metrics reported in Results. Traits are ordered by average performance. Scientific surveys (SCI) and combined datasets (COMB) perform similarly well, while citizen science data alone (CIT) shows consistently lower accuracy and higher variability, highlighting the importance of structured ecological surveys for reliable trait predictions at large scales. Source data are available at `https://zenodo.org/records/18108765`.

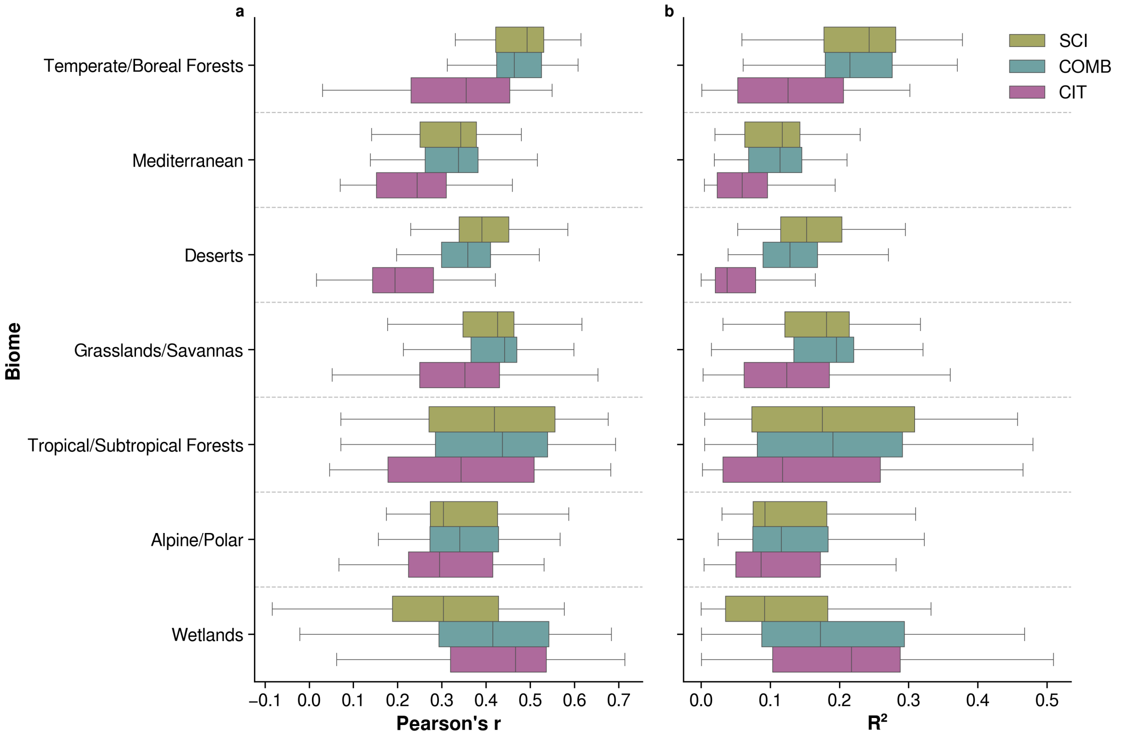

Figure S.3: **Model performance across biomes for trait prediction at 1 km^2 resolution.** Boxplots show the distribution of (a) Pearson correlation coefficients (r) and (b) coefficients of determination (R^2) for trait predictions across different biomes (n=31 trait models). Three trait data subsets are compared: SCI (vegetation survey data only, olive), COMB (combined vegetation survey and citizen science data, teal), and CIT (citizen science data only, magenta). Each box represents the interquartile range with the median shown as a horizontal line within each box. Whiskers extend to 1.5 times the interquartile range, with individual points representing outliers. Results are shown for models using power-transformed traits at 1 km^2 spatial resolution. Horizontal dashed lines separate biome groups for visual clarity. The combined trait data subset (COMB) generally shows slight to moderate improved performance compared to individual data sources across most biomes. Source data are available at `https://zenodo.org/records/18108765`.

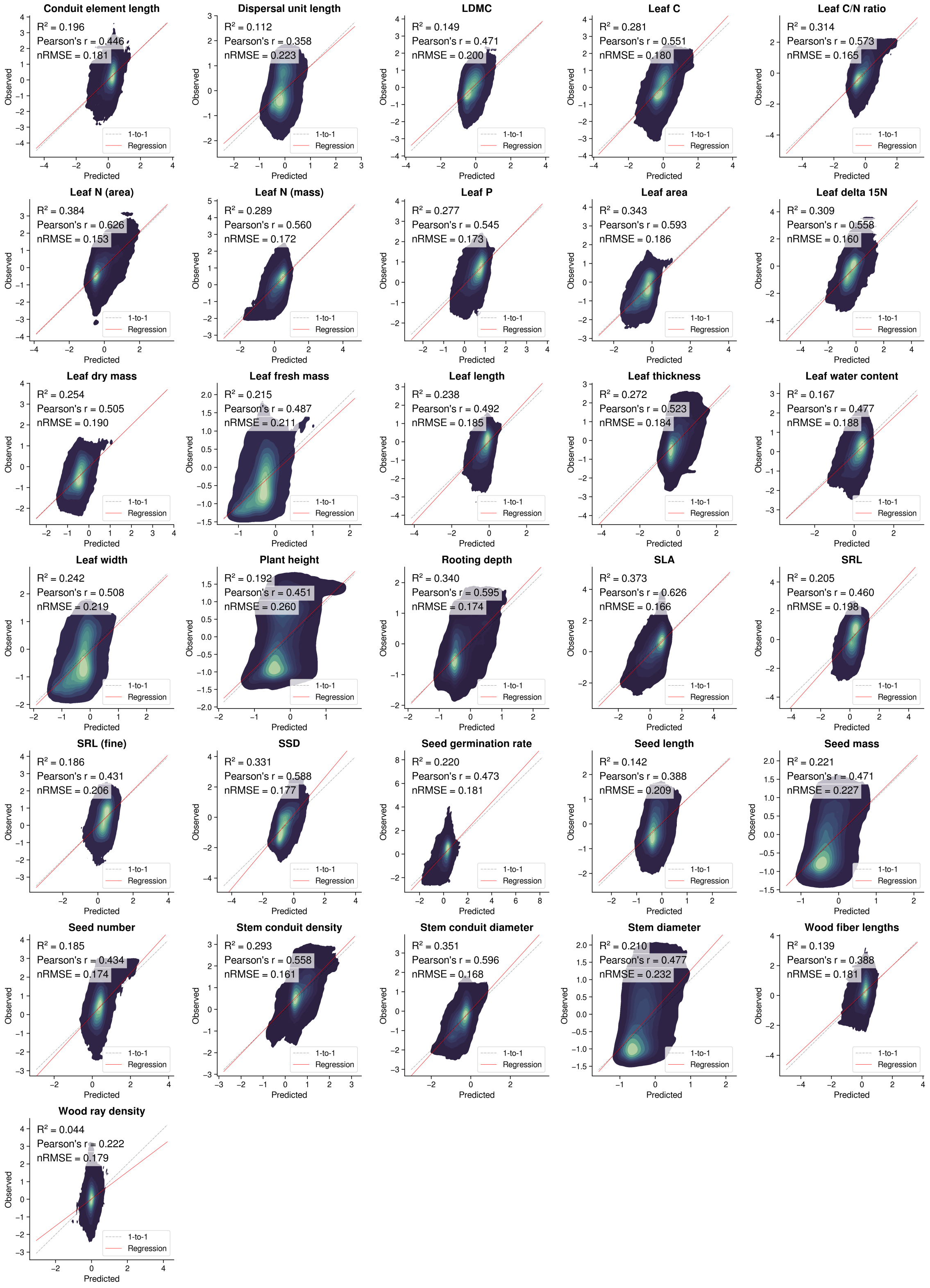

Figure S.4: **Observed versus predicted community-weighted mean trait values from models trained on vegetation survey and citizen science data (COMB).** Scatter plots are presented as density plots, with lighter colors representing higher point density. Model performance statistics are shown, as well as a grey dashed 1-to-1 line representating a correlation of 1 and a linear regression line in solid red. All trait values are in Yeo-Johnson power-transformed space. Source data are available at `https://zenodo.org/records/18108765`.

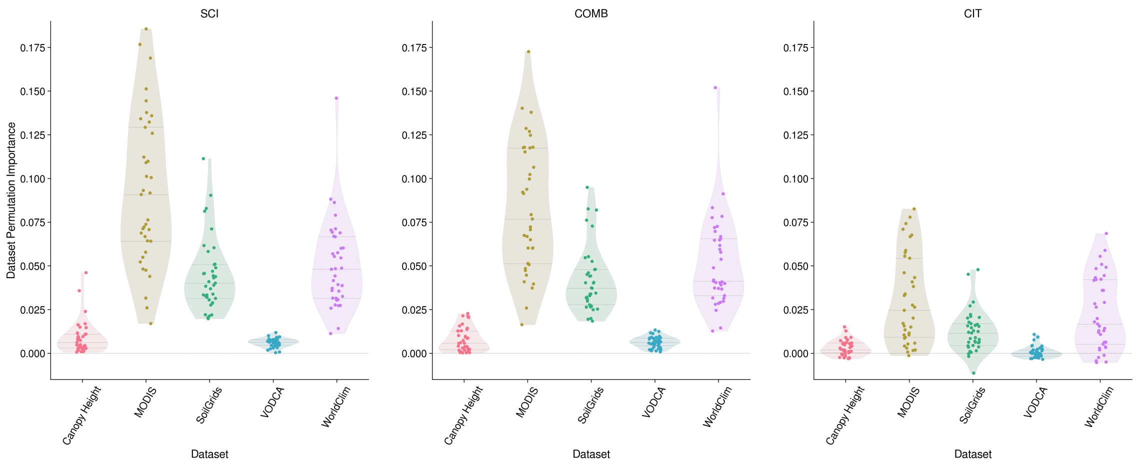

Figure S.5: **Dataset permutation importance of each dataset used in model training.** Here, dataset permutation importance refers to the effect of randomly shuffling the values of *all* features within a given dataset on trait model performance. Individual dots represent traits (n=31 trait models), and each plot represents a different trait data subset: vegetation survey data alone (SCI, left), citizen science data alone (CIT, right), and a combination of both (COMB, middle). Source data are available at `https://zenodo.org/records/18108765`.

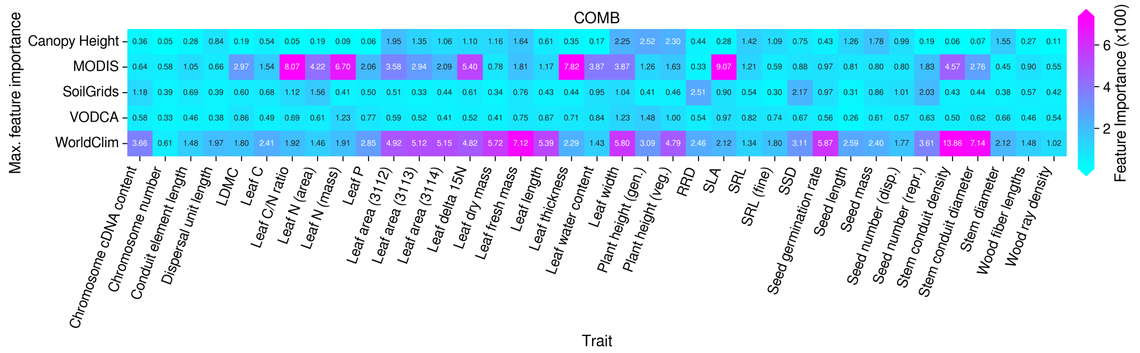

Figure S.6: **Maximum feature permutation importance of each dataset used in COMB model training.** Here, maximum feature permutation importance refers to the maximum permutation importance of a feature within each dataset for each trait model. Values have been multiplied by 100 for readability. Source data are available at `https://zenodo.org/records/18108765`.

Supplementary Tables

Table S.1: **Impact of plant functional type (PFT) matching on trait data availability from the TRY database.** Numbers show species richness and observation counts before and after filtering, with retention percentages in parentheses.

Trait Name	Species (Before)	Species (After)	Observations (Before)	Observations (After)
Conduit element length	53,025	34,957 (65.9%)	927,121	803,655 (86.7%)
Dispersal unit length	55,300	35,990 (65.1%)	933,493	811,155 (86.9%)
LDMC	49,903	33,122 (66.4%)	928,363	808,923 (87.1%)
Leaf C	51,616	33,905 (65.7%)	926,093	803,050 (86.7%)
Leaf C/N ratio	50,612	33,564 (66.3%)	926,069	806,531 (87.1%)
Leaf N (area)	46,127	31,214 (67.7%)	930,895	811,494 (87.2%)
Leaf N (mass)	51,115	33,858 (66.2%)	926,399	808,332 (87.3%)
Leaf P	51,343	33,634 (65.5%)	929,944	807,846 (86.9%)
Leaf area	49,668	33,435 (67.3%)	930,573	812,583 (87.3%)
Leaf delta 15N	52,760	34,484 (65.4%)	927,482	805,544 (86.9%)
Leaf dry mass	48,121	32,795 (68.2%)	933,805	816,176 (87.4%)
Leaf fresh mass	48,387	32,972 (68.1%)	932,088	814,395 (87.4%)
Leaf length	52,657	34,658 (65.8%)	930,467	807,966 (86.8%)
Leaf thickness	50,749	33,666 (66.3%)	927,686	807,278 (87.0%)
Leaf water content	49,037	32,752 (66.8%)	928,445	810,099 (87.3%)
Leaf width	51,352	34,171 (66.5%)	928,809	808,376 (87.0%)
Plant height	53,370	34,592 (64.8%)	915,844	798,391 (87.2%)
Rooting depth	51,725	34,060 (65.8%)	936,725	813,433 (86.8%)
SLA	46,596	31,524 (67.7%)	930,708	811,952 (87.2%)
SRL	53,612	35,154 (65.6%)	937,029	812,880 (86.8%)
SRL (fine)	53,459	35,092 (65.6%)	932,919	810,168 (86.8%)
SSD	55,849	36,208 (64.8%)	925,431	796,733 (86.1%)
Seed germination rate	54,380	35,386 (65.1%)	920,859	797,266 (86.6%)
Seed length	57,361	37,062 (64.6%)	935,612	813,911 (87.0%)
Seed mass	61,548	39,187 (63.7%)	932,520	810,023 (86.9%)
Seed number	51,567	34,063 (66.1%)	934,358	812,032 (86.9%)
Stem conduit density	52,698	34,539 (65.5%)	931,116	805,450 (86.5%)
Stem conduit diameter	53,491	34,893 (65.2%)	926,438	801,594 (86.5%)
Stem diameter	54,446	35,175 (64.6%)	918,142	794,485 (86.5%)
Wood fiber lengths	52,222	34,543 (66.1%)	925,953	803,688 (86.8%)
Wood ray density	53,411	35,054 (65.6%)	932,862	806,657 (86.5%)

Table S.2: **Model performance statistics across resolutions and trait data subsets.** The "best" values per trait per resolution are indicated in bold, representing the highest values for R^2 and r and the lowest values for nRMSE. The top performing models for each trait across all resolutions are indicated with an asterisk (*). Resolutions are in square kilometers.

		R^2					r					nRMSE				
		1 km	22 km	55 km	111 km	222 km	1 km	22 km	55 km	111 km	222 km	1 km	22 km	55 km	111 km	222 km
**Conduit element length**	SCI	**0.204**	**0.268**	**0.275***	0.233	0.189	**0.454**	**0.518**	**0.524***	0.485	0.436	**0.180**	**0.153**	**0.150***	0.153	0.160
	COMB	0.196	0.265	0.269	**0.241**	**0.205**	0.446	0.515	0.519	**0.492**	**0.455**	0.181	0.154	0.151	**0.152**	**0.158**
	CIT	0.108	0.112	0.111	0.112	0.092	0.341	0.341	0.341	0.340	0.309	0.190	0.169	0.166	0.165	0.169
**Dispersal unit length**	SCI	**0.116**	**0.172**	**0.216**	0.232	**0.335***	**0.374**	**0.434**	**0.477**	0.490	**0.580***	**0.222**	**0.200**	**0.187**	0.191	**0.178***
	COMB	0.112	0.161	0.200	**0.233**	0.326	0.358	0.418	0.457	**0.491**	0.574	0.223	0.201	0.189	**0.190**	0.179

Continued on next page

Table S.2: **(Cont.) Model performance statistics across resolutions and trait data subsets.** The "best" values per trait per resolution are indicated in bold, representing the highest values for R^2 and r and the lowest values for nRMSE. The top performing models for each trait across all resolutions are indicated with an asterisk (*). Resolutions are in square kilometers.

		**R²**					**r**					**nRMSE**				
		1 km	22 km	55 km	111 km	222 km	1 km	22 km	55 km	111 km	222 km	1 km	22 km	55 km	111 km	222 km
	CIT	-0.089	-0.242	-0.136	-0.060	0.027	0.227	0.140	0.272	0.337	0.404	0.247	0.245	0.225	0.224	0.215
**LDMC**	SCI	**0.246***	**0.234**	**0.213**	**0.159**	**0.084**	**0.498***	**0.487**	**0.467**	**0.411**	**0.328**	**0.188**	**0.185**	**0.181***	**0.194**	**0.201**
	COMB	0.149	0.133	0.125	0.099	0.033	0.471	0.429	0.428	0.379	0.326	0.200	0.196	0.191	0.201	0.206
	CIT	-0.406	-0.702	-0.827	-0.872	-1.036	0.377	0.311	0.276	0.244	0.261	0.256	0.275	0.276	0.290	0.299
**Leaf C**	SCI	**0.316**	**0.408**	**0.410***	**0.370**	**0.367**	**0.564**	**0.639**	**0.641***	**0.612**	**0.608**	**0.175**	**0.164**	**0.163**	**0.162***	**0.165**
	COMB	0.281	0.357	0.372	0.339	0.351	0.551	0.615	0.619	0.594	0.602	0.180	0.171	0.168	0.166	0.167
	CIT	0.010	-0.138	-0.141	-0.126	-0.072	0.394	0.354	0.364	0.361	0.381	0.211	0.228	0.227	0.216	0.215
**Leaf C/N ratio**	SCI	**0.337**	**0.399***	**0.387**	**0.318**	**0.243**	**0.581**	**0.632***	**0.623**	**0.565**	**0.497**	**0.163**	**0.160***	**0.161**	**0.166**	**0.173**
	COMB	0.314	0.366	0.343	0.286	0.215	0.573	0.622	0.608	0.559	0.491	0.165	0.164	0.167	0.170	0.176
	CIT	0.112	-0.084	-0.179	-0.247	-0.229	0.497	0.491	0.473	0.421	0.424	0.188	0.215	0.223	0.225	0.220
**Leaf N (area)**	SCI	**0.397**	**0.461***	**0.426**	0.354	**0.346**	**0.631**	**0.679***	0.653	0.596	0.592	**0.151***	**0.151***	**0.154**	**0.161**	**0.168**
	COMB	0.384	0.448	0.423	**0.359**	0.345	0.626	0.676	**0.655**	**0.605**	**0.593**	0.153	0.153	0.155	**0.161**	**0.168**
	CIT	0.223	0.121	0.138	0.117	0.117	0.547	0.529	0.537	0.522	0.515	0.172	0.193	0.189	0.189	0.195
**Leaf N (mass)**	SCI	**0.322**	**0.398***	**0.356**	**0.282**	**0.206**	**0.568**	**0.632***	**0.598**	0.532	**0.469**	**0.168**	**0.161***	**0.170**	**0.176**	**0.181**
	COMB	0.289	0.355	0.303	0.266	0.179	0.560	0.616	0.586	**0.540**	0.462	0.172	0.167	0.177	0.178	0.184
	CIT	0.009	-0.196	-0.306	-0.359	-0.451	0.488	0.486	0.472	0.439	0.371	0.203	0.227	0.242	0.242	0.245
**Leaf P**	SCI	**0.280**	**0.380**	**0.406***	**0.364**	**0.365**	0.533	**0.617**	**0.638***	**0.608**	0.607	**0.172**	**0.165***	**0.171**	**0.183**	**0.186**
	COMB	0.277	0.315	0.360	0.314	0.320	**0.545**	0.586	0.618	0.594	0.587	0.173	0.173	0.177	0.190	0.192
	CIT	0.141	0.059	0.095	0.043	0.048	0.512	0.572	0.619	0.606	**0.631**	0.188	0.203	0.211	0.225	0.228
**Leaf area**	SCI	**0.346**	**0.468**	**0.523**	**0.537**	0.553	0.590	**0.684**	**0.723**	0.733	0.744	**0.186**	**0.156**	**0.142***	**0.146**	0.149
	COMB	0.343	0.448	0.503	0.536	**0.556***	**0.593**	0.674	0.714	**0.735**	**0.746***	**0.186**	0.159	0.145	**0.146**	**0.148**
	CIT	0.179	0.215	0.290	0.353	0.414	0.538	0.608	0.651	0.671	0.704	0.208	0.189	0.173	0.172	0.170
**Leaf delta 15N**	SCI	0.308	**0.407**	**0.456***	0.423	0.381	0.557	**0.640**	**0.675***	0.651	0.622	**0.160**	**0.152**	**0.149***	0.155	0.156
	COMB	**0.309**	0.404	0.447	**0.433**	**0.407**	**0.558**	0.636	0.673	**0.660**	**0.640**	**0.160**	**0.152**	0.150	**0.154**	**0.152**
	CIT	0.221	0.227	0.260	0.289	0.324	0.504	0.550	0.583	0.601	0.629	0.170	0.173	0.174	0.172	0.163
**Leaf dry mass**	SCI	0.243	**0.425**	**0.487**	0.478	0.506	0.498	**0.652**	**0.698**	0.691	0.713	0.192	**0.156**	**0.146***	**0.154**	0.162
	COMB	**0.254**	0.396	0.465	**0.480**	**0.524***	**0.505**	0.630	0.683	**0.693**	**0.724***	**0.190**	0.160	0.149	**0.154**	**0.159**
	CIT	0.147	0.212	0.305	0.384	0.436	0.408	0.470	0.559	0.623	0.663	0.204	0.183	0.170	0.167	0.173
**Leaf fresh mass**	SCI	0.208	**0.376**	**0.434**	0.434	0.518	0.466	**0.617**	**0.661**	0.665	0.724	0.212	**0.173**	**0.160***	0.169	0.168
	COMB	**0.215**	0.365	0.415	**0.451**	**0.533***	**0.487**	0.605	0.648	**0.672**	**0.730***	**0.211**	0.174	0.162	**0.166**	**0.165**
	CIT	0.129	0.221	0.265	0.378	0.475	0.451	0.512	0.575	0.639	0.706	0.222	0.193	0.182	0.177	0.175
**Leaf length**	SCI	0.221	**0.338**	**0.360**	0.328	0.398	0.474	**0.582**	**0.601**	0.577	**0.637***	0.187	**0.171**	**0.165**	0.166	**0.158***
	COMB	**0.238**	0.336	**0.360**	**0.372**	**0.402***	**0.492**	**0.582**	**0.601**	**0.610**	0.636	**0.185**	**0.171**	**0.165**	**0.160**	**0.158***
	CIT	0.167	0.192	0.263	0.280	0.338	0.423	0.477	0.548	0.554	0.597	0.193	0.189	0.177	0.171	0.166
**Leaf thickness**	SCI	**0.286**	0.285	**0.255**	0.169	0.158	**0.540**	0.537	**0.512**	0.429	0.426	**0.182**	0.180	**0.180**	0.185	0.183
	COMB	0.272	**0.299***	0.247	**0.183**	**0.169**	0.523	**0.551***	0.506	**0.439**	**0.433**	0.184	**0.178***	0.181	**0.184**	**0.182**
	CIT	0.159	0.012	-0.017	-0.029	0.003	0.419	0.308	0.310	0.299	0.325	0.198	0.211	0.210	0.206	0.199
**Leaf water content**	SCI	**0.257***	**0.247**	**0.204**	**0.141**	0.069	**0.507***	**0.500**	**0.458**	**0.390**	0.312	**0.178**	**0.176***	**0.177**	**0.189**	**0.196**
	COMB	0.167	0.169	0.115	0.102	**0.078**	0.477	0.456	0.412	0.369	**0.326**	0.188	0.185	0.187	0.194	**0.196**
	CIT	-0.406	-0.635	-0.793	-0.825	-0.924	0.370	0.309	0.287	0.259	0.302	0.245	0.260	0.266	0.276	0.282
**Leaf width**	SCI	**0.254**	**0.358**	**0.391**	**0.390**	0.462	**0.508**	**0.600**	**0.626**	**0.628**	0.683	**0.217**	**0.185**	**0.174***	**0.180**	0.181
	COMB	0.242	0.333	0.375	0.386	**0.467***	**0.508**	0.587	0.614	0.623	**0.685***	0.219	0.189	0.177	0.181	**0.180**
	CIT	0.152	0.130	0.192	0.242	0.355	0.484	0.524	0.573	0.600	0.665	0.231	0.216	0.201	0.201	0.198
**Plant height**	SCI	**0.210**	**0.278***	**0.278***	**0.227**	0.230	**0.488**	**0.542***	**0.538**	**0.495**	0.497	**0.257**	**0.246***	**0.246***	**0.255**	0.258
	COMB	0.192	0.236	0.266	0.207	**0.237**	0.451	0.507	0.531	0.494	**0.505**	0.260	0.254	0.248	0.258	**0.257**
	CIT	-0.072	-0.292	-0.264	-0.221	-0.115	0.289	0.179	0.213	0.261	0.363	0.299	0.330	0.326	0.320	0.311
**Rooting depth**	SCI	**0.383**	**0.387***	**0.348**	**0.318**	0.338	**0.619**	**0.622***	**0.593**	0.566	0.585	**0.168***	**0.174**	**0.183**	**0.193**	0.194
	COMB	0.340	0.352	0.331	**0.318**	**0.341**	0.595	0.604	0.585	**0.569**	**0.586**	0.174	0.179	0.185	**0.193**	**0.193**
	CIT	0.051	-0.011	0.024	0.051	0.163	0.406	0.406	0.432	0.459	0.536	0.208	0.224	0.223	0.227	0.218
**SLA**	SCI	**0.394**	**0.457***	**0.426**	**0.355**	**0.281**	**0.630**	**0.676***	**0.653**	**0.598**	**0.538**	**0.163***	**0.169**	**0.172**	**0.177**	**0.178**
	COMB	0.373	0.397	0.372	0.321	0.233	0.626	0.651	0.632	0.586	0.519	0.166	0.178	0.180	0.182	0.184
	CIT	0.124	-0.183	-0.291	-0.338	-0.414	0.529	0.451	0.437	0.420	0.410	0.196	0.249	0.259	0.255	0.250
**SRL**	SCI	**0.236**	**0.339***	**0.315**	0.260	0.149	**0.489**	**0.583***	**0.564**	**0.520**	0.412	**0.194**	**0.178***	**0.182**	0.189	0.193
	COMB	0.205	0.297	0.299	**0.262**	**0.153**	0.460	0.556	0.554	0.517	**0.417**	0.198	0.183	0.184	**0.188**	**0.192**
	CIT	0.016	-0.176	-0.204	-0.219	-0.185	0.189	0.032	0.042	0.018	0.070	0.221	0.237	0.241	0.242	0.227
**SRL (fine)**	SCI	**0.189**	**0.233**	**0.257***	0.211	0.152	**0.439**	**0.483**	**0.508***	0.462	0.412	**0.205**	**0.179**	**0.176***	**0.183**	0.188
	COMB	0.186	0.212	0.246	**0.213**	**0.191**	0.431	0.463	0.499	**0.467**	**0.443**	0.206	0.182	0.178	**0.183**	**0.184**
	CIT	0.072	0.022	0.055	0.029	0.048	0.280	0.281	0.331	0.324	0.358	0.219	0.202	0.199	0.203	0.199
**SSD**	SCI	**0.350**	**0.383***	**0.372**	0.306	0.290	**0.592**	**0.619***	**0.611**	0.560	**0.548**	**0.175**	**0.160***	**0.162**	0.175	**0.183**
	COMB	0.331	0.353	0.353	**0.315**	**0.291**	0.588	0.600	0.599	**0.569**	**0.548**	0.177	0.163	0.165	**0.174**	**0.183**
	CIT	0.193	0.126	0.160	0.138	0.150	0.494	0.467	0.505	0.501	0.522	0.195	0.190	0.188	0.195	0.200
**Seed germination rate**	SCI	**0.242**	**0.398***	**0.391**	0.296	0.222	**0.495**	**0.631***	**0.626**	0.547	0.478	**0.179**	**0.156***	**0.157**	0.171	0.172
	COMB	0.220	0.357	0.380	**0.317**	**0.231**	0.473	0.610	0.621	**0.567**	**0.490**	0.181	0.161	0.158	**0.169**	**0.171**
	CIT	0.061	-0.017	-0.057	-0.095	-0.076	0.301	0.375	0.355	0.281	0.284	0.199	0.203	0.206	0.214	0.202

Continued on next page

Table S.2: **(Cont.) Model performance statistics across resolutions and trait data subsets.** The "best" values per trait per resolution are indicated in bold, representing the highest values for R^2 and r and the lowest values for nRMSE. The top performing models for each trait across all resolutions are indicated with an asterisk (*). Resolutions are in square kilometers.

		**R²**					**r**					**nRMSE**				
		1 km	22 km	55 km	111 km	222 km	1 km	22 km	55 km	111 km	222 km	1 km	22 km	55 km	111 km	222 km
**Seed length**	SCI	**0.144**	**0.235**	**0.331**	0.357	0.403	**0.392**	**0.489**	**0.577**	0.604	0.641	**0.209**	**0.180**	**0.165***	0.168	0.170
	COMB	0.142	0.203	0.316	**0.369**	**0.438***	0.388	0.458	0.565	**0.610**	**0.662***	**0.209**	0.183	0.167	**0.166**	**0.165***
	CIT	-0.027	-0.018	0.118	0.207	0.283	0.265	0.342	0.468	0.530	0.582	0.229	0.207	0.189	0.186	0.186
**Seed mass**	SCI	**0.223**	**0.297**	0.349	0.379	**0.420***	**0.477**	**0.548**	0.595	0.618	**0.650***	**0.227**	**0.190**	0.180	0.180	**0.180**
	COMB	0.221	0.275	**0.356**	**0.389**	0.418	0.471	0.527	**0.598**	**0.624**	0.647	**0.227**	0.193	**0.179**	**0.178***	**0.180**
	CIT	0.114	0.125	0.236	0.272	0.316	0.354	0.402	0.508	0.538	0.573	0.242	0.212	0.195	0.194	0.195
**Seed number**	SCI	**0.211**	**0.455***	**0.423**	0.373	0.351	**0.459**	**0.674***	**0.650**	0.612	0.599	**0.171**	**0.141***	**0.147**	0.149	0.153
	COMB	0.185	0.447	0.413	**0.389**	**0.361**	0.434	0.670	0.647	**0.625**	**0.603**	0.174	0.142	0.149	**0.147**	**0.152**
	CIT	0.037	0.020	0.059	0.083	0.091	0.201	0.227	0.306	0.350	0.350	0.189	0.189	0.188	0.181	0.182
**Stem conduit density**	SCI	**0.308**	**0.410**	**0.477**	**0.523***	0.501	**0.564**	0.644	**0.691**	0.723	0.711	**0.159**	**0.151**	**0.147***	**0.147***	0.155
	COMB	0.293	0.394	0.456	0.520	**0.511**	0.558	**0.646**	0.685	**0.724**	0.719	0.161	0.153	0.150	**0.147***	**0.153**
	CIT	0.052	0.039	0.147	0.231	0.343	0.469	0.594	0.657	0.686	**0.728***	0.186	0.192	0.187	0.186	0.178
**Stem conduit diameter**	SCI	0.350	0.461	**0.502**	**0.521**	0.578	0.592	0.680	0.709	0.722	0.762	**0.168**	0.151	**0.144**	**0.145**	0.142
	COMB	**0.351**	**0.471**	0.499	**0.521**	**0.595***	**0.596**	**0.688**	**0.710**	**0.724**	**0.772***	**0.168**	**0.149**	0.145	**0.145**	**0.139***
	CIT	0.241	0.259	0.312	0.367	0.463	0.523	0.601	0.649	0.679	0.742	0.182	0.176	0.170	0.167	0.160
**Stem diameter**	SCI	**0.254**	**0.286**	**0.318***	**0.264**	**0.257**	**0.507**	**0.536**	**0.565***	**0.520**	**0.518**	**0.226**	**0.208***	**0.208***	**0.219**	**0.224**
	COMB	0.210	0.228	0.287	0.253	0.255	0.477	0.499	0.541	0.508	0.516	0.232	0.217	0.212	0.220	**0.224**
	CIT	-0.019	-0.176	-0.126	-0.098	-0.001	0.287	0.228	0.299	0.348	0.438	0.264	0.267	0.267	0.267	0.259
**Wood fiber lengths**	SCI	**0.160**	**0.239***	**0.216**	0.176	0.113	**0.402**	**0.488***	**0.466**	0.423	0.355	**0.179**	**0.159**	**0.162**	**0.157***	0.165
	COMB	0.139	0.233	0.208	**0.183**	**0.139**	0.388	0.484	0.459	**0.432**	**0.384**	0.181	0.160	0.163	**0.157***	**0.163**
	CIT	-0.022	-0.049	-0.066	-0.072	-0.079	0.217	0.136	0.135	0.131	0.122	0.197	0.187	0.189	0.179	0.182
**Wood ray density**	SCI	0.041	**0.070**	**0.107**	**0.141**	0.159	**0.228**	**0.282**	**0.344**	**0.382**	0.408	**0.179**	**0.171**	**0.167**	**0.163***	0.170
	COMB	**0.044**	0.066	0.100	0.122	**0.169***	0.222	0.267	0.327	0.358	**0.412***	**0.179**	**0.171**	**0.167**	0.165	**0.169**
	CIT	-0.013	-0.011	0.033	0.049	0.071	0.116	0.095	0.190	0.223	0.269	0.184	0.178	0.173	0.172	0.179

Table S.3: **Pearson correlation between sPlot and GBIF sparse CWM trait grids.** Resolutions are in square kilometers.

	1 km	22 km	55 km	111 km	222 km
Chromosome cDNA content	0.20	0.14	0.14	0.18	0.21
Chromosome number	0.22	0.17	0.18	0.24	0.27
Conduit element length	0.25	0.29	0.28	0.29	0.32
Dispersal unit length	0.26	0.20	0.28	0.36	0.46
LDMC	0.33	0.28	0.27	0.27	0.27
Leaf C	0.44	0.27	0.30	0.33	0.32
Leaf C/N ratio	0.46	0.41	0.42	0.45	0.44
Leaf N (area)	0.41	0.50	0.49	0.53	0.54
Leaf N (mass)	0.44	0.42	0.43	0.44	0.44
Leaf P	0.51	0.55	0.61	0.64	0.64
Leaf area	0.44	0.56	0.63	0.69	0.72
Leaf area (3112)	0.43	0.56	0.63	0.69	0.72
Leaf area (3114)	0.47	0.57	0.64	0.70	0.73
Leaf delta 15N	0.46	0.44	0.53	0.57	0.62
Leaf dry mass	0.37	0.45	0.55	0.63	0.68
Leaf fresh mass	0.34	0.48	0.56	0.64	0.70
Leaf length	0.39	0.41	0.49	0.56	0.62
Leaf thickness	0.37	0.34	0.31	0.33	0.33
Leaf water content	0.29	0.28	0.27	0.28	0.29
Leaf width	0.38	0.48	0.56	0.63	0.67
Plant height	0.31	0.20	0.26	0.33	0.42
Plant height (gen.)	0.29	0.21	0.28	0.35	0.43
Rooting depth	0.27	0.35	0.40	0.48	0.55
SLA	0.48	0.45	0.41	0.42	0.43
SRL	0.28	0.11	0.11	0.13	0.18
SRL (fine)	0.34	0.24	0.30	0.36	0.41
SSD	0.46	0.41	0.44	0.48	0.51
Seed germination rate	0.19	0.25	0.29	0.31	0.33
Seed length	0.28	0.31	0.44	0.52	0.59
Seed mass	0.37	0.35	0.46	0.54	0.61
Seed number	0.23	0.18	0.23	0.29	0.35
Seed number (disp.)	0.29	0.29	0.31	0.32	0.34
Stem conduit density	0.37	0.51	0.63	0.69	0.74
Stem conduit diameter	0.39	0.53	0.63	0.71	0.76
Stem diameter	0.33	0.22	0.28	0.35	0.44
Wood fiber lengths	0.20	0.16	0.13	0.16	0.18
Wood ray density	0.18	0.08	0.13	0.17	0.21

Table S.4: **Original and aggregated biome definitions.** Biome definitions were extracted from the Terrestrial Ecoregions of the World dataset by Olson *et al.* [1] and used for assessing model performance and spatial transferability across biomes. Mean trait observation counts per biome are shown for GBIF and sPlot datasets (mean ± standard deviation).

**Biome Set**	**Original Biome from TEOW**	**n (GBIF)**	**n (sPlot)**
Tropical/Subtropical Forests	Tropical & Subtropical Moist Broadleaf Forests	$1.25(\pm0.02)\times10^5$	$4.23(\pm0.51)\times10^3$
	Tropical & Subtropical Dry Broadleaf Forests		
	Tropical & Subtropical Coniferous Forests		
Temperate/Boreal Forests	Temperate Broadleaf & Mixed Forests	$1.55(\pm0.01)\times10^6$	$2.52(\pm0.26)\times10^5$
	Temperate Conifer Forests		
	Boreal Forests/Taiga		
Grasslands/Savannas	Tropical & Subtropical Grasslands, Savannas & Shrublands	$1.57(\pm0.03)\times10^5$	$1.41(\pm0.24)\times10^4$
	Temperate Grasslands, Savannas & Shrublands		
Mediterranean	Mediterranean Forests, Woodlands & Scrub	$3.34(\pm0.04)\times10^5$	$3.43(\pm0.53)\times10^4$
Deserts	Deserts and Xeric Shrublands	$7.07(\pm0.37)\times10^4$	$2.53(\pm0.32)\times10^4$
Wetlands	Flooded Grasslands and Savannas	$1.03(\pm0.02)\times10^4$	$2.16(\pm0.22)\times10^2$
	Mangroves		
	Lake		
Alpine/Polar	Montane Grasslands and Shrublands	$5.83(\pm0.04)\times10^4$	$5.72(\pm0.53)\times10^3$
	Tundra		
	Rock and Ice		

References

1. Olson, D. M. *et al.* Terrestrial Ecoregions of the World: A New Map of Life on Earth: A new global map of terrestrial ecoregions provides an innovative tool for conserving biodiversity. *BioScience* **51,** 933–938. doi:10.1641/0006-3568(2001)051[0933:TEOTWA]2.0.CO;2 (2001).
