# Supplementary material for: Crowdsourced biodiversity monitoring fills gaps in global plant trait mapping
Source: Nat Commun. 2026 Jan 30;17:1203. doi: 10.1038/s41467-026-68996-y (PMC12858941; doi:10.1038/s41467-026-68996-y)
Supplement: Supplementary file 2 — Reporting Summary [file 41467_2026_68996_MOESM2_ESM.pdf]

nature portfolio

Corresponding author(s): Daniel Lusk

Last updated by author(s): Dec 31, 2025

Reporting Summary

Nature Portfolio wishes to improve the reproducibility of the work that we publish. This form provides structure for consistency and transparency in reporting. For further information on Nature Portfolio policies, see our Editorial Policies and the Editorial Policy Checklist.

Statistics

For all statistical analyses, confirm that the following items are present in the figure legend, table legend, main text, or Methods section.

n/a	Confirmed	
☐	☒	The exact sample size (*n*) for each experimental group/condition, given as a discrete number and unit of measurement
☐	☒	A statement on whether measurements were taken from distinct samples or whether the same sample was measured repeatedly
☐	☒	The statistical test(s) used AND whether they are one- or two-sided<br>*Only common tests should be described solely by name; describe more complex techniques in the Methods section.*
☐	☒	A description of all covariates tested
☐	☒	A description of any assumptions or corrections, such as tests of normality and adjustment for multiple comparisons
☐	☒	A full description of the statistical parameters including central tendency (e.g. means) or other basic estimates (e.g. regression coefficient) AND variation (e.g. standard deviation) or associated estimates of uncertainty (e.g. confidence intervals)
☒	☐	For null hypothesis testing, the test statistic (e.g. *F*, *t*, *r*) with confidence intervals, effect sizes, degrees of freedom and *P* value noted<br>*Give P values as exact values whenever suitable.*
☒	☐	For Bayesian analysis, information on the choice of priors and Markov chain Monte Carlo settings
☒	☐	For hierarchical and complex designs, identification of the appropriate level for tests and full reporting of outcomes
☐	☒	Estimates of effect sizes (e.g. Cohen's *d*, Pearson's *r*), indicating how they were calculated

Our web collection on statistics for biologists contains articles on many of the points above.

Software and code

Policy information about availability of computer code

Data collection	No software was used to collect the data in this study.
Data analysis	Data analysis was performed using open-source libraries with the Python language ecosystem. Raster data management was performed using rasterio (v1.4.1) and xarray (v2024.9.0). Dataframe management was performed using pandas (v2.2.2) and dask (v2024.9.0). Statistical analysis was performed using scipy (v1.12.0), scikit-learn (v1.4.2), and pykrige (v1.7.2). Machine learning model training was performed using lightgbm (v4.5.0) and imputation was performed using verstack (v4.1.4). All code, versioning, and example Jupyter notebooks can be found at https://github.com/GeoSense-Freiburg/cit-sci-traits.

For manuscripts utilizing custom algorithms or software that are central to the research but not yet described in published literature, software must be made available to editors and reviewers. We strongly encourage code deposition in a community repository (e.g. GitHub). See the Nature Portfolio guidelines for submitting code & software for further information.

Data

Policy information about availability of data

All manuscripts must include a data availability statement. This statement should provide the following information, where applicable:

- Accession codes, unique identifiers, or web links for publicly available datasets
- A description of any restrictions on data availability
- For clinical datasets or third party data, please ensure that the statement adheres to our policy

The Earth observation data used in this study are publicly available through Google Earth Engine (https://earthengine.google.com) or the Google Earth Engine

Community Catalog (https://gee-community-catalog.org/). GBIF species occurrence data are available via the dataset citation provided in the References \cite {gbiforg_user_occurrence_2024}.

The TRY gapfilled trait data used in this study are available under restricted access due to the data sharing policies of contributing datasets within TRY. Access can be requested through the TRY Plant Trait Database (https://www.try-db.org) following their standard data request procedure.

The sPlot vegetation survey data used in this study are available under restricted access to protect the interests of data contributors. Access can be requested by contacting the sPlot consortium through the German Centre for Integrative Biodiversity Research (iDiv) via G.D. or through the sPlot website (https://www.idiv.de/splot). After request processing, data are provided under a data use agreement.

The global trait maps generated in this study have been deposited in Zenodo (https://doi.org/10.5281/zenodo.14646321). An interactive map viewer is available at https://global-traits.projects.earthengine.app/view/global-traits, and additional study resources can be found at https://planttraits.earth. Users of these maps should consult the coefficient of variation and area of applicability layers, as well as the model performance metrics provided in the raster metadata and Table S.2, noting that performance varies across traits and biomes (Figs.4b, 5c, S.3; Table S.4).

Source data are provided with this paper. All Source Data used for the generation of figures and tables can be found at https://doi.org/10.5281/zenodo.18108765 (source data) and https://doi.org/10.5281/zenodo.14646321 (final outputs).

Research involving human participants, their data, or biological material

Policy information about studies with human participants or human data. See also policy information about sex, gender (identity/presentation), and sexual orientation and race, ethnicity and racism.

Reporting on sex and gender	*Use the terms sex (biological attribute) and gender (shaped by social and cultural circumstances) carefully in order to avoid confusing both terms. Indicate if findings apply to only one sex or gender; describe whether sex and gender were considered in study design; whether sex and/or gender was determined based on self-reporting or assigned and methods used. Provide in the source data disaggregated sex and gender data, where this information has been collected, and if consent has been obtained for sharing of individual-level data; provide overall numbers in this Reporting Summary. Please state if this information has not been collected. Report sex- and gender-based analyses where performed, justify reasons for lack of sex- and gender-based analysis.*
Reporting on race, ethnicity, or other socially relevant groupings	*Please specify the socially constructed or socially relevant categorization variable(s) used in your manuscript and explain why they were used. Please note that such variables should not be used as proxies for other socially constructed/relevant variables (for example, race or ethnicity should not be used as a proxy for socioeconomic status). Provide clear definitions of the relevant terms used, how they were provided (by the participants/respondents, the researchers, or third parties), and the method(s) used to classify people into the different categories (e.g. self-report, census or administrative data, social media data, etc.) Please provide details about how you controlled for confounding variables in your analyses.*
Population characteristics	*Describe the covariate-relevant population characteristics of the human research participants (e.g. age, genotypic information, past and current diagnosis and treatment categories). If you filled out the behavioural & social sciences study design questions and have nothing to add here, write "See above."*
Recruitment	*Describe how participants were recruited. Outline any potential self-selection bias or other biases that may be present and how these are likely to impact results.*
Ethics oversight	*Identify the organization(s) that approved the study protocol.*

Note that full information on the approval of the study protocol must also be provided in the manuscript.

Field-specific reporting

Please select the one below that is the best fit for your research. If you are not sure, read the appropriate sections before making your selection.

☐ Life sciences ☐ Behavioural & social sciences ☒ Ecological, evolutionary & environmental sciences

For a reference copy of the document with all sections, see nature.com/documents/nr-reporting-summary-flat.pdf

Ecological, evolutionary & environmental sciences study design

All studies must disclose on these points even when the disclosure is negative.

Study description	Our study introduces and investigates a large-scale data integration approach to modeling and mapping 31 ecologically-relevant plant functional traits at high resolution and global extent. We combine three crowdsourced plant biodiversity monitoring efforts to generate community-weighted mean (CWM) trait values as reference data, and we model each of the 31 traits as a function of 150 Earth observation predictors at spatial resolutions from 1 km to 222 km. We evaluate the resulting maps against spatially independent, held-out CWM trait values, compare performance with previous trait mapping studies, and assess the influence of citizen science data on model spatial transferability.
Research sample	For reference data, we used three crowdsourced plant biodiversity monitoring efforts: vegetation survey data from the sPlot database, individual vascular plant observations from the Global Biodiversity Information Facility (GBIF), and in situ trait

measurements from the TRY trait database. sPlot survey data was obtained from Francesco Maria Sabatini of the sPlot consortium with express permission of both the data curators and contributors. GBIF data was retrieved directly from gbif.org. The traits of interest for the study were retrieved directly from Jens Kattge of TRY (IDs: 4, 6, 13, 14, 15, 21, 26, 27, 46, 47, 50, 55, 78, 95, 138, 144, 145, 146, 163, 169, 237, 281, 282, 289, 297, 614, 1080, 3106, 3113, 3117, 3120). All Earth observation data used is openly accessible and was retrieved either using Google Earth Engine or from the respective dataset webpage.

Sampling strategy

All available vegetation plots containing species relative cover from the sPlot database were used. All non-cultivated vascular plant observations containing no likely geotagging errors from the GBIF database were used, and during spatial aggregation grid cells containing fewer than 10 observations were discarded. Grid cells containing greater than GBIF 500 observations were randomly sampled to retain a maximum of 500 observations. All available trait measurements for all available species in the TRY trait database were used. To perform spatial cross-validation for each trait model, the autocorrelation range of the sPlot-derived community-weighted mean (CWM) trait values was calculated. Spatially independent folds were then assigned based on the spatial autocorrelation range, and each fold was held out during cross-validation.

Data collection

The GBIF vascular plant database consists of observations dating back to the 1600s, contributed by a myriad of observers including, in large part, citizen scientists using the iNaturalist application since 2008. Vegetation surveys in sPlot and trait measurements in TRY were conducted by a multitude of scientists, researchers, and volunteers around the world. Earth observation data was made available both through remote sensing campaigns from major national space programs including NASA and ESA as well as significant modeling efforts by environmental and climate researchers.

Timing and spatial scale

All observations from sPlot, GBIF, and TRY were used, spanning nearly 400 years of data collection. After matching with TRY, sPlot and GBIF observations were then gridded using mean aggregation to 1 km, 22 km, 55 km, 111 km, and 222 km spatial resolutions. All MODIS surface reflectance observations from March 2020-present were aggregated into 12 monthly means. All VODCA observations, from 1987-2018 were retrieved and aggregated into three statistics for each band: 5th percentile, mean, and 95th percentile. All data included observations at the broadest spatial extent available, and where sufficient predictor and/or reference data was available. All Earth observation predictors with the exception of VODCA were available natively at finer or equal to 1 km resolution. For spatial scales of 1 km and 22 km, VODCA data were upscaled from 0.25 degree resolution to the respective resolutions using bilinear interpolation.

Data exclusions

GBIF observations marked as "cultivated" or that contained likely georeferencing errors were excluded. Otherwise, no data were excluded.

Reproducibility

All code has been made available, and the data versioning and the pipeline tool Data Version Control (dvc.org) was utilized to make pipeline reproduction as simple as possible. Additionally, Jupyter notebooks were created to provide a step-by-step documentation of the analysis. While not all data used is directly publicly accessible (e.g. sPlot and TRY gap-filled measurements), all datasets do have an open-access analogue. sPlot data is available as sPlotOpen, and non-gap-filled TRY trait measurements can be obtained from trydb.org. Source Data has also been made available for the reproduction of figures and tables.

Randomization

This study utilized continuous trait data and therefore data was not grouped and did not require randomization.

Blinding

This study used existing data and therefore data acquisition was performed independently of the study. No blinding was required.

Did the study involve field work? ☐ Yes ☒ No

Reporting for specific materials, systems and methods

We require information from authors about some types of materials, experimental systems and methods used in many studies. Here, indicate whether each material, system or method listed is relevant to your study. If you are not sure if a list item applies to your research, read the appropriate section before selecting a response.

Materials & experimental systems

n/a	Involved in the study
☒	☐ Antibodies
☒	☐ Eukaryotic cell lines
☒	☐ Palaeontology and archaeology
☒	☐ Animals and other organisms
☒	☐ Clinical data
☒	☐ Dual use research of concern
☒	☐ Plants

Methods

n/a	Involved in the study
☒	☐ ChIP-seq
☒	☐ Flow cytometry
☒	☐ MRI-based neuroimaging

Plants

Seed stocks

Report on the source of all seed stocks or other plant material used. If applicable, state the seed stock centre and catalogue number. If plant specimens were collected from the field, describe the collection location, date and sampling procedures.

Novel plant genotypes

Describe the methods by which all novel plant genotypes were produced. This includes those generated by transgenic approaches, gene editing, chemical/radiation-based mutagenesis and hybridization. For transgenic lines, describe the transformation method, the number of independent lines analyzed and the generation upon which experiments were performed. For gene-edited lines, describe the editor used, the endogenous sequence targeted for editing, the targeting guide RNA sequence (if applicable) and how the editor was applied.

Authentication

Describe any authentication procedures for each seed stock used or novel genotype generated. Describe any experiments used to assess the effect of a mutation and, where applicable, how potential secondary effects (e.g. second site T-DNA insertions, mosiacism, off-target gene editing) were examined.
